# A Rare Case of Eccrine Carcinoma Demonstrating Intracranial Invasion in a Patient With Advanced Hepatocellular Carcinoma

**DOI:** 10.7759/cureus.63760

**Published:** 2024-07-03

**Authors:** Haroon A Dossani, Omayr M Irshad, Bassel Ibrahim, Billie Shine, Jayati Mallick, Arsalan Saleem

**Affiliations:** 1 Vascular and Interventional Radiology, University of Texas Medical Branch at Galveston, Galveston, USA; 2 Pathology, University of Texas Medical Branch at Galveston, Galveston, USA

**Keywords:** eccrine porocarcinoma (epc), tumor-free resection margins, metachronous mass, intracranial invasion, hepatocellular carcinoma (hcc), eccrine carcinoma (ec)

## Abstract

Eccrine carcinoma (EC) is a rare intraepidermal carcinoma of the eccrine sweat glands. Even more rare are instances of EC exhibiting intracranial invasion. Here, we describe the case of a metachronous EC mass demonstrating intracranial invasion in a patient with advanced-stage hepatocellular carcinoma (HCC), reporting CT head findings of a left frontal skull expansile destructive mass with soft tissue density and immunostain findings of the following: CEA: positive, granular, EMA: positive, AE1/AE3: positive, CK7: strongly positive, CK20: negative, GCDFP: negative, and HEPAR: negative. The only recommended treatment for EC is surgical excision with tumor-free margins, and no chemotherapy protocols currently exist. Due to socioeconomic factors, our patient was unable to receive adequate treatment for her HCC, nor surgical excision for her EC. However, the unique presentation of a rare intracranial EC tumor causing no neurological deficits in a patient with untreated HCC merits the need for a more thorough identification of secondary tumors via biopsy in patients with HCC to identify possible associations between these two tumors in future patients.

## Introduction

Eccrine carcinoma (EC) is an intraepidermal carcinoma of the eccrine sweat gland typically seen in older patients, although it can present in any age group [1]. The first case was documented in 1971 [2], and since then there have been very few documented cases. EC lesions typically occur in the legs (60% of all cases), usually occurring on the thigh and knee [1]. The average age of onset is 60 years old [3] and prognosis is generally poor, with relative mortality for metastatic disease at 80%, and a 9% 10-year disease overall survival rate [4]. EC tumors are characterized as locally aggressive with a high rate of recurrence [4]. There are limited previous reports of eccrine carcinoma with scalp invasion, with only two previous cases being identified in the literature [2,5]. Here we describe a rather rare case of scalp eccrine carcinoma demonstrating intracranial invasion to contribute to the list of cases in the medical literature.

Upon physical inspection, eccrine carcinomas usually appear as solitary lesions in the upper and lower extremities (14% and 60% of the time, respectively) and the head and neck (24% of the time) [3,4]. It is worth noting that EC is a rare tumor and is often misdiagnosed due to its relatively low incidence. By utilizing both ultrasound and MR imaging, a more accurate diagnosis of EC can be made, leading to more targeted and effective treatment [6].

Ultrasound

Ultrasound imaging of EC shows a well-defined, multilobulated lesion located in the dermis and subcutaneous tissue of the affected area. The lesion displayed hypoechoic and heterogeneous solid components with increased peripheral vascularity, as well as cystic components containing echoic spots. These ultrasound findings are important in diagnosing EC and can help in distinguishing it from other malignant tumors such as malignant melanomas or basal cell carcinomas that are identifiable with low area under curve on ultrasound [7,8].

CT

Eccrine carcinoma is difficult to diagnose on CT due to presenting as nonspecific soft tissue density with poor soft tissue contrast. Therefore, CT in the case of EC is primarily useful for the detection of bone invasion and metastasis [8].

MRI

Because eccrine carcinomas usually appear as solitary lesions, a narrow field of view is recommended for the MRI protocol. The axial plane is preferred due to its specificity in identifying involved adjacent tissue, and an additional plane should also be obtained [8]. The most important MRI sequences for diagnosis are T1WI, T2WI, and fat-suppressed T2WI, each serving a different clinical purpose. T1WI is useful for determining subcutaneous fat invasion and whether the tumor contains fat or hemorrhage. T2WI provides information on signal intensity and tumor cellularity while fat-suppressed T2WI detects peritumoral fat stranding and adjacent tissue invasion. MR imaging can be useful in diagnosing EC, with MR findings of eccrine porocarcinoma (EPC) including a pedunculated configuration, homogenous T2 signal, hyperintensity in T1 weighted images, and the presence of cystic components [7].

18F-FDG-PET/CT

Flourine-18 fluorodeoxyglucose positron emission tomography/computed tomography (18F-FDG-PET/CT is used in the case of EC to detect metastases, both nodal and distant, as well as bone invasion. Furthermore, it can be used to detect micrometastases and subtle recurrences in instances where the patient is already diagnosed with EC [8].

Although both CT and MRI are adequate imaging forms for EC, MRIs are preferred due to their superior specificity and clear imaging of tumor size, characteristics, and local invasion. Given the high sensitivity but low specificity of MRI imaging for this specific tumor, it is recommended to use it as an aid in diagnosis and as follow-up imaging for tumor treatment. Biopsies are typically required to confirm diagnoses since low signal intensities on T1WIs and intermediate to high intensities on T2WI are not specific for eccrine carcinomas, with the exception of lipomas [9].

Differential diagnoses

The differential diagnoses to be considered in the case of EC include Paget’s disease, basal cell carcinoma, melanoma, inflammatory/lymphocytic/vascular lesions, and metastatic cancer [10].

## Case presentation

Description of case

**Figure 1:**
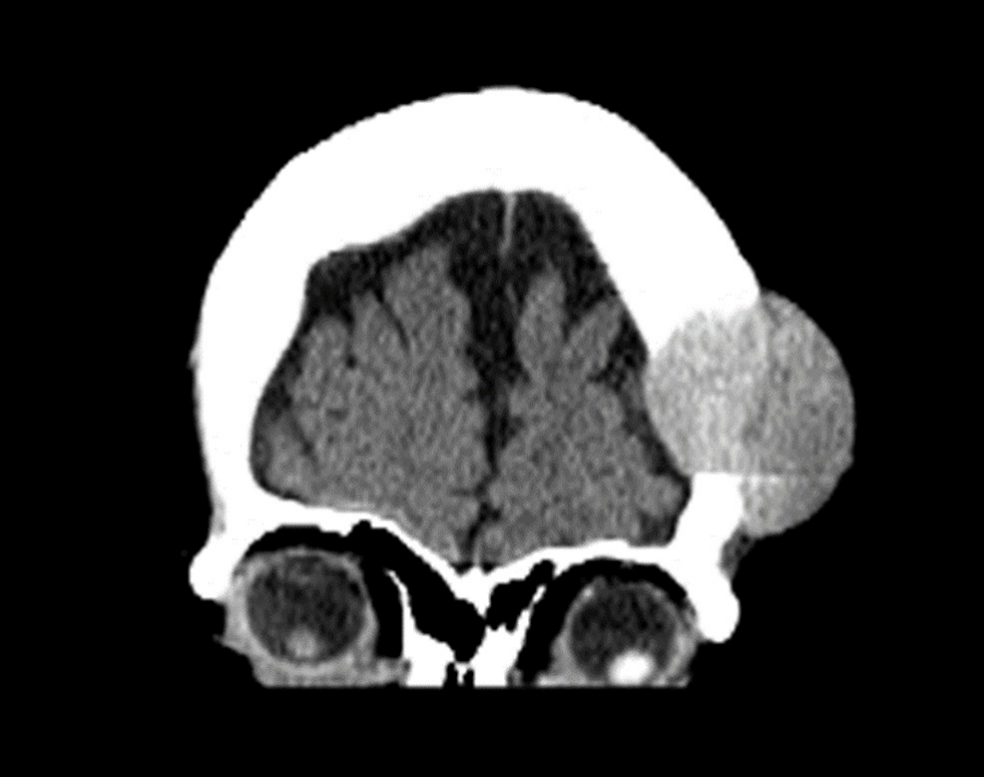
CT head of mass (coronal view)

**Figure 2:**
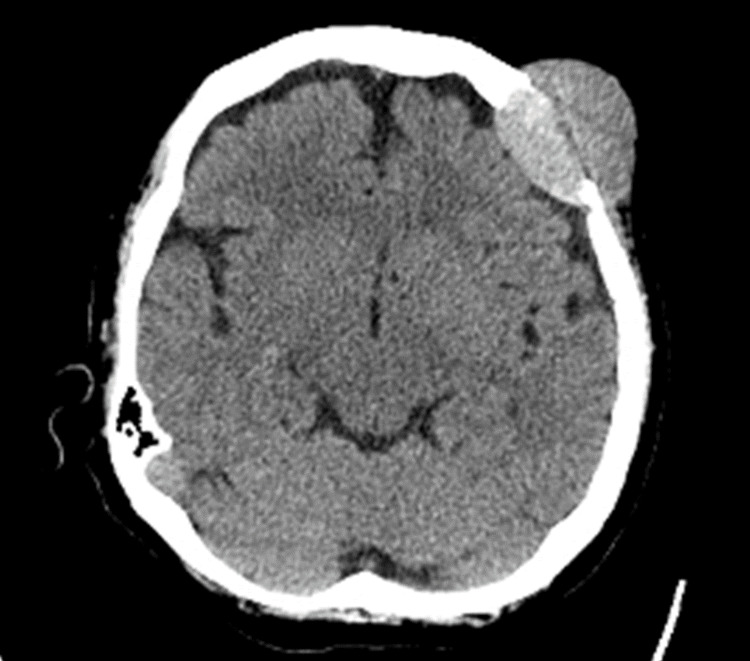
CT head of mass (axial view)

The following case is of a 63-year-old woman with a past medical history of hepatitis C virus (HCV), chronic liver disease, liver cirrhosis, and advanced-stage hepatocellular carcinoma (HCC). She had not received any treatment for her HCC, despite having been diagnosed over 18 months ago, due to a lack of health insurance and inconsistent medical care. The patient presented to a primary care clinic following a fall, where the examiner found a mass on her head and suspected it to be a brain metastasis of her HCC. Upon referral to neurology, the patient had no neurological symptoms and the mass appeared extradural. A referral was made to radiology and non-contrast CT imaging of the head revealed a 4.2 cm x 3.5 cm x 4.7 cm (AP x TR x CC) left frontal skull expansile destructive mass with soft tissue density. Below are the CT findings with coronal and axial views (Figures 1, 2).

**Figure 3:**
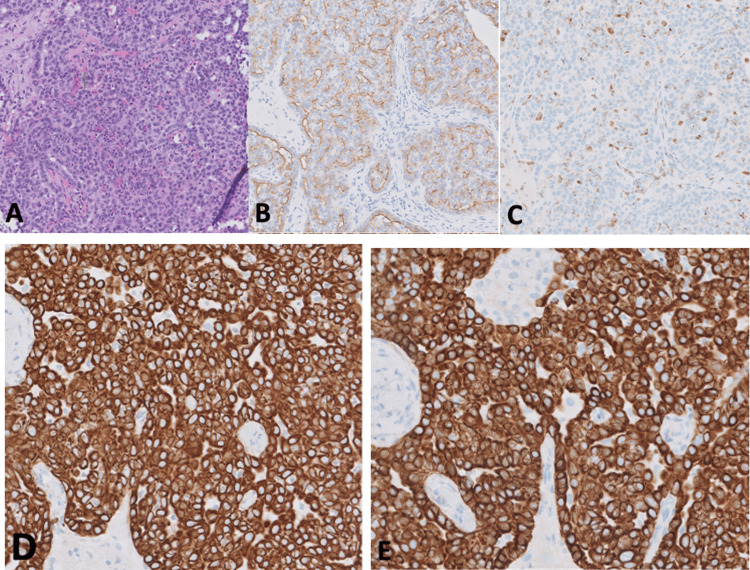
Pathology of eccrine carcinoma biopsy with H&E stained initial sample (a) with the following immunostains performed: (b) positive EMA, (c) positive CEA, (d) positive AE1/AE3, (e) strongly positive CK7 and negative CK20 H&E- hematoxylin and eosin; EMA- epithelial membrane antigen; CEA- carcinoembryonic antigen; AE1/AE3- Cocktail of two different anti-cytokeratin monoclonal antibodies; CK7- cytokeratin 7; CK19- cytokeratin 19

Following the identification of a left frontal skull mass in our patient, an ultrasound-guided percutaneous left frontal calvarial biopsy of the mass revealed multiple tan-white soft tissue core fragments ranging from 0.2 cm to 1.4 cm (1.4 x 0.4 x 0.1 cm in aggregate). Immunostain results showed cells that were CEA positive and granular, EMA positive, AE1/AE3 positive, strongly positive for CK7, negative for CK20, negative for GCDFP, and negative for HEPAR. Figure 3 shows the results of the biopsy.

Ultimately, the patient passed away 18 months after the initial diagnosis of her HCC due to severe metabolic encephalopathy, for which her family opted for comfort care.

## Discussion

A biopsy was needed to identify the skull mass, as other differential diagnoses for a head mass of this kind include intraosseous meningioma and hemangiopericytoma, which can both present as similar head masses on radiology. CT findings for intraosseous meningioma include a hyperattenuated homogenous lytic mass [11]. MRI findings for intraosseous meningioma include an isointense biconvex mass on a precontrast axial T1 image, with intense homogenous enhancement and mild dural reaction on postcontrast axial T1 MRI [11]. CT findings for hemangiopericytoma include heterogeneously hyperdense tumors with focal areas of hypodensity and do enhance with contrast [12]. MRI findings for hemangiopericytoma include isointensity to cortical gray matter on both T1 and T2 weighted imaging, and heterogeneous enhancement with contrast on T1WI [12].

The recommended treatment of eccrine tumors is surgical excision with tumor-free margins, with no treatment standards for the use of chemotherapy or radiation therapy in place [1]. However, if dural venous sinus involvement is present, surgical resection is no longer a viable treatment option [2]. In this case, the extradural eccrine carcinoma mass was not surgically excised due to the concern of surgical excision delaying systemic treatment for her hepatocellular carcinoma.

Although we did not remove the mass, we present this case to draw two important points to the attention of medical literature. First, the occurrence of a metachronous, second primary mass in a patient with HCC is interesting because one would expect this second mass to be a metastasis given the patient’s advanced HCC. This was the conclusion of multiple physicians who saw the patient until a biopsy was done but, given the diagnosis of eccrine carcinoma following the biopsy, there is a need for a more thorough examination of secondary tumors to see if there is any correlation between HCC and eccrine carcinoma. There is currently no known syndromic association between HCC and EC, but future cases should be monitored to determine if there is a correlation. Second, an eccrine mass displaying intracranial invasion causing no neurological deficits is rare and has no treatment protocols besides excision. Although our patient was unable to receive proper medical treatment for both this mass and her HCC due to inconsistent medical care and a lack of insurance, treatment efforts and protocols should be published for other patients presenting with intracranial eccrine carcinomas to try and develop treatment, specifically chemotherapy, protocols.

## Conclusions

EC with intracranial invasion is shown to better resolve with aggressive surgical resection if treated early on. However, due to the close involvement of venous sinuses, this procedure may be contraindicated due to the possibility of heavy bleeding. Thus, patients with little to no treatment may present with extracranial metastases. The best prognosis with EC would be early detection with MRI to determine tumor margins and limited FDG-avid foci on FDG-PET/CT to suggest distant metastases. Curative measures would involve radical tumor resection including all involved lymph nodes followed by short-term follow-ups with imaging to evaluate for any recurrence.

In the case of this patient, resection may have reduced and confined the margins of the tumor, but recurrence would have been highly likely and her mean survival rate would not have improved. However, her HCC was a greater determinant of her overall prognosis, and addressing the metachronous EC was secondary.

## References

[1] Ahn CS, Sangüeza OP (2019). Malignant sweat gland tumors. Hematol Oncol Clin North Am.

[2] Kibe Y, Tanahashi K, Ohtakara K (2022). Direct intracranial invasion of eccrine spiradenocarcinoma of the scalp: a case report and literature review. BMC Neurol.

[3] Staiger RD, Helmchen B, Papet C, Mattiello D, Zingg U (2017). Spiradenocarcinoma: a comprehensive data review. Am J Dermatopathol.

[4] Kaseb H, Babiker HM (2022). Eccrine carcinoma. Diagnostic Pathology: Neoplastic Dermatopathology.

[5] Pedamallu SB, Murphy J, Boyd D, Martin-Hirsch D, Al-Zwae K (2009). Direct intracranial extension of malignant eccrine spiradenoma of the face. J Clin Med Res.

[6] Tsiogka A, Koumaki D, Kyriazopoulou M, Liopyris K, Stratigos A, Gregoriou S (2023). Eccrine porocarcinoma: a review of the literature. Diagnostics (Basel).

[7] Mahdar I, Lembarki G, Jamil J (2022). Eccrine porocarcinoma: an extremely rare cutaneous tumor from a radiological point of view - case report and review of the literature. BJR Case Rep.

[8] Kawaguchi M, Kato H, Noda Y, Kobayashi K, Miyazaki T, Hyodo F, Matsuo M (2022). Imaging findings of malignant skin tumors: radiological-pathological correlation. Insights Imaging.

[9] Park E, Lee DS, Eom KS (2015). Eccrine poroma on the scalp: a case report with MR findings. Nerve.

[10] Idrissi Serhrouchni K, Harmouch T, Chbani L (2013). Eccrine carcinoma: a rare cutaneous neoplasm. Diagn Pathol.

[11] Agrawal V, Ludwig N, Agrawal A, Bulsara KR (2007). Intraosseous intracranial meningioma. AJNR Am J Neuroradiol.

[12] Chiechi M V., Smirniotopoulos JG, Mena H (1996). Intracranial hemangiopericytomas: MR and CT features. AJNR Am J Neuroradiol.

